# Personal Experience

**Published:** 1910-03-15

**Authors:** L. P. Haskell


### PERSONAL EXPERIENCE.

BY DR. L. P. HASKELL.

Dental practice is flooded with theories, some proved correct but practically of no account, others never proved but still urged.

Personal experience, however, is very potent sometimes in the settlement of so-called theories.

A personal experience of twelve years in the use of a full lower denture has very forcibly revealed to me some things not otherwise attainable.

One problem of the dentist is the excessive absorption of the lower jaw until, in my own case, there is but four-sixteenths of an inch of bone left in the region of the first molars. In addition to this, there is often a flexible condition of membrane over a considerable portion of the jaw. A plate constructed for such a jaw, I care not what the impression is, will not prove at once comfortable, when the pressure of the muscles is brought to bear upon it. There will be irritation at various points that must be relieved, for it is impossible to masticate otherwise. In such cases it will be weeks before perfect comfort and usefulness is acquired.

I say that no dentist who has not had this personal experience in his own mouth has any idea of what patients often have to contend with and too much effort cannot be spared to inform the patient of what they may have to experience and to impress upon them the necessity of going to the dentist for relief at once in case of irritation.

Why there should be such excessive absorption of the lower jaw I never have been able to understand. It proceeds regardless of what material is worn upon it, in fact when no plate has been worn after the loss of the posterior teeth.

In these cases there needs to be retoration where the process is lost in order to restore the contour of the lips. This makes a long bite, which necessarily increases leverage in mastication, making the plate more difficult to use for a time.

In this class of cases it must be borne in mind that although there may be some depression on the lingual side of the jaw, the plate cannot be worn below the margin at all, because it will be lifted not only by the muscles, but by a mass of glands and loose membrane. Even laying the end of my finger firmly a very little over the margin upon lifting the tongue the finger is displaced.

It is just here that many patients are enduring unnecessary annoyance because the dentist has thoughtlessly extended the plate below the margin.

For many years I recommended the use of cast metal plates. The second plate I made for myself was one of them. After having them in for a short time I went into the laboratory and leaned forward to speak to a student and the denture slid forward, because on this flat jaw there was nothing to resist the weight. That night, when lying down, it slid into the cheek, so I had no further use for it.

Of course, if there is a ridge to straddle that trouble would not occur, but my experience has fully demonstrated to me that a vulcanite plate is just as good upon any lower jaw as anything that is worn, and that weight is not a necessity in any case.

I have found that a flange on each side is an advantage in that the cheeks hold the plate down and keep it from moving forward.

In relieving the irritated spot, although it is readily seen, it is not always easy to locate it on the plate. Take a little moist whiting on a spatula and touch the spot, and upon replacing the plate it is readily seen.

Where the posterior teeth have long been removed and a plate worn and later on the anterior teeth are removed and a full plate worn, as the anterior gums settle and the bite shortens at this point, the posterior teeth do not shorten, and under pressure there results irritation at the heel. The remedy is found in shortening the molars and not in filing the plate.—*Review*, May, 1908.

